# Sustainable Palm Oil—The Role of Screening and Advanced Analytical Techniques for Geographical Traceability and Authenticity Verification

**DOI:** 10.3390/molecules25122927

**Published:** 2020-06-25

**Authors:** Umi Salamah Ramli, Noor Idayu Tahir, Nurul Liyana Rozali, Abrizah Othman, Nor Hayati Muhammad, Syahidah Akmal Muhammad, Azmil Haizam Ahmad Tarmizi, Norfadilah Hashim, Ravigadevi Sambanthamurthi, Rajinder Singh, Mohamad Arif Abd Manaf, Ghulam Kadir Ahmad Parveez

**Affiliations:** 1 Malaysian Palm Oil Board, No. 6 Persiaran Institusi, Bandar Baru Bangi, Kajang 43000, Selangor, Malaysia; idayu@mpob.gov.my (N.I.T.); liyana@mpob.gov.my (N.L.R.); abi@mpob.gov.my (A.O.); ati@mpob.gov.my (N.H.M.); azmil_haizam@mpob.gov.my (A.H.A.T.); dila@mpob.gov.my (N.H.); ravigadevi@gmail.com (R.S.); rajinder@mpob.gov.my (R.S.); arifma@mpob.gov.my (M.A.A.M.); parveez@mpob.gov.my (G.K.A.P.); 2 School of Industrial Technology/Analytical Biochemistry Research Centre, Universiti Sains Malaysia, USM, George Town 11800, Penang, Malaysia; syahidah.muhammad@usm.my

**Keywords:** palm oil, sustainability, geographical traceability, adulteration, analytical techniques, chemical fingerprint, DNA fingerprint

## Abstract

Palm oil production from oil palm (*Elaeis guineensis* Jacq.) is vital for the economy of Malaysia. As of late, sustainable production of palm oil has been a key focus due to demand by consumer groups, and important progress has been made in establishing standards that promote good agricultural practices that minimize impact on the environment. In line with the industrial goal to build a traceable supply chain, several measures have been implemented to ensure that traceability can be monitored. Although the palm oil supply chain can be highly complex, and achieving full traceability is not an easy task, the industry has to be proactive in developing improved systems that support the existing methods, which rely on recorded information in the supply chain. The Malaysian Palm Oil Board (MPOB) as the custodian of the palm oil industry in Malaysia has taken the initiative to assess and develop technologies that can ensure authenticity and traceability of palm oil in the major supply chains from the point of harvesting all the way to key downstream applications. This review describes the underlying framework related to palm oil geographical traceability using various state-of-the-art analytical techniques, which are also being explored to address adulteration in the global palm oil supply chain.

## 1. Introduction

Palm oil is extracted from the mesocarp of oil palm (*Elaeis guineensis* Jacq.), which belongs to the family (Arecaceae), and is of West African origin. Malaysia and Indonesia are responsible for 85% of the global production of palm oil, which accounts for 34% of the world’s vegetable oil requirement [1]. This makes South East Asia the most important region responsible for supplying the oils and fats required by the world’s growing population. The oil palm fruit contains two kinds of oil, palm oil from the fleshy outer layer, and palm kernel oil from the seed. Palm oil contains 44% palmitic acid (C16:0) as the major saturated fatty acid, counter balanced by the unsaturated fatty acids, which include oleic acid (~39%) and linoleic acid (~11%). The composition of palm kernel oil is almost 80% saturated [2,3]. The different fatty acid (FA) profiles of palm oil and palm kernel oil allow for their varied applications in the downstream industry. The identity characteristics and quality requirements of Malaysian palm oil as specified under the revised National Standard MS814:2007 (2018) is provided in Table 1.

The specifications relate to the mesocarp oil from *Elaeis guineensis*, which is the main commercial species planted in Asia, Africa, and South America [5]. A second interfertile species, *Elaeis oleifera*, which originates from South America, has higher unsaturated mesocarp oil contributed by elevated levels of oleic acid (C18:1) (~59%) and linoleic acid (C18:2) (~19%) with much reduced content of the saturated palmitic acid (C16:0) (~17%) when compared to *E. guineensis* [6]. The lower yield profiles of *E. oleifera* make it unattractive for commercial cultivation. However, resistance to disease, especially bud rot, has generated interest in *E. oleifera*, especially in South America, where the disease is widespread. Interspecific hybrids, which have intermediate yield and oil unsaturation levels of both species but inherit the resistance from *E. oleifera*, are the only materials suitable for planting in areas devastated by the disease in South America [5].

Food industries consume 80% of palm oil and the remaining 20% is used for industrial (non-food) applications such as in the biodiesel industry [7]. Its diverse applications in food and non-food including biofuels and its competitive price in comparison to other vegetable oils, make palm oil an appealing and attractive commodity [8,9,10,11]. The increase in world population, expected to reach 9.15 billion by 2050 [12], will also raise the requirement for vegetable oils, estimated at 240 million tonnes in the same period [13]. Being the most productive oil crop, delivering three to eight times more oil per unit area of crop land compared to other temperate or tropical oil crops, oil palm is well positioned to meet the growing demand for oils and fats in a sustainable manner. As the most traded commodity, palm oil contributes about 5% to 6% to Malaysia’s Gross Domestic Product (GDP) [14]. It generated RM 64.84 billion in earnings in 2019 as compared to RM 67.52 billion in 2018 (Table 2).

The exports of Malaysian palm oil are mainly concentrated in five major markets such as India, China, the European Union (EU), Pakistan, and the United States of America (U.S.A), comprising nearly 60% of total exports [1,7,16]. In 2019, Malaysia exported a total of 2.09 million tonnes of palm oil to the EU-up 9.5% from 1.91 million tonnes recorded in 2018-and accounting for 11.3% of Malaysia’s total 2019 palm oil exports. However, the European Parliament resolution of 4th April 2017 decreed that, due to concern on oil palm’s impact on the environment and biodiversity, palm oil will be phased out by the EU for use as transport fuel in 2021 [1,17,18,19]. This decision was made even though EU had made a commitment to purchase only certified sustainable palm oil that is traceable from the year 2020 onwards. Although the desire to phase out palm oil for use as biodiesel in the EU has now been postponed to a later date, this raises the possibility that, apart from EU, other importing countries may also impose similar conditions, where only “certified sustainable and traceable” palm oil would be purchased for use in their country.

Pressure from international groups, especially those from the EU, have created the desire within the oil palm industry to ensure the practices are transparent and meet the desired sustainability standards. Both governments and non-governmental organisations (NGO) have established a number of regulations over time to ensure the long-term viability of the industry and that it meets the triple bottom line, which include the 3Ps (people, planet, and profit) of sustainable development. The oil palm industry aspires to ensure that business profitability (profit) does not compromise the interest of society (people), and that there is a minimum impact on the environment (planet). In fact, the oil palm sector is one of the few industries that actively encourages its members to join certification schemes such as the Malaysian Sustainable Palm Oil (MSPO) [20,21] and Roundtable on Sustainable Palm Oil (RSPO) [22,23] as the first global standard setting initiative. This is aimed at responsibly producing palm oil that is traceable across the supply chain, as a firm commitment by the industry to adhere to the triple bottom line concept, which will ensure that the commodity continues to be an important pillar of the Malaysian economy [1]. In order to ensure traceability across the supply chain, ideally sustainably produced palm oil that has met RSPO or MSPO standards has to be kept physically separate from non-certified produced palm oil from the point of production to the port and until the stage it reaches consumers. There has to be a mechanism in place that can ensure traceability to the source of origin or to detect any attempts at adulteration with non-certified palm oil or any other sources at any point in the supply chain.

Currently, the traceability measures in place are not robust enough to provide reliable and conclusive analytical evidence that the palm oil is procured from a sustainable source certified by schemes such as RSPO or MSPO if such proof is ever required. The current measures in place for certification are based on documentation, which relies on input given by specific producers. Accuracy of the information is sometimes difficult to verify and there is a general agreement within the industry that a more robust and reliable set-up is required to provide a transparent traceability system. Since the EU will only acquire “certified sustainable palm oil” from a verifiable source, it is important to guarantee that the oils are obtained from the proclaimed source, ideally by means of proven analytical methods [19]. An effective traceability system that accurately tracks a product and provides accurate information regarding its origin, across the supply chain, adds value and will enhance profitability [24,25]. At present, the accreditation and certification procedures of sustainable palm oil depend on administrative controls and assessments.

Apparently, the analytical methods currently available are not suitable for routine application to discriminate palm oil from the sustainable and non-sustainable sources. Such methods include classical physiochemical tests to ascertain characteristics such as apparent density, refractive index, slip melting point, iodine value (IV), saponification value, peroxide value, and fatty acid composition. These classical methods, while relevant for quality analysis, are often time consuming, expensive, and are not suitable for routine application for traceability, especially those involving a large number of samples or batches, and require highly trained personnel. The ability to discriminate palm oil from different regions/sources will require the development of a geographical traceability system by utilizing robust and accurate methods that allow the palm oil consignment to be traced through every production step back to its origin. Promising markers that could facilitate the differentiation of palm oil include stable isotope signatures, fatty acid profiles, phytosterols, carotenoids, and volatile organic compounds in combination with chemometrics [19,26,27]. This will ensure that traceability in the supply chain can be monitored using appropriate analytical tools. This will add value to the current certification system in place used to determine if the palm oil being traded has been produced in a sustainable and eco-friendly manner. The strengths and shortcomings of existing methods used to discriminate vegetable oils, such as olive oil, based on their geographical origin was summarized [28]. This analysis is a good starting point, where the available information can facilitate the development of appropriate analytical tools for establishing a traceability system for palm oil across its supply chain. In line with this, studies carried out on olive oil demonstrated the feasibility of characterizing the triacylglycerol and chemical composition of its oil as an effective means to differentiate olive oil cultivated in different geographical regions [29,30,31]. Most of the techniques applied used various analytical tools to determine if the product was produced from a determined geographical area, including the protected designation of origin (PDO). The PDO products were differentiated based on (1) volatile compounds [32], (2) tocopherols, sterols, and fatty acid composition [33], (3) trace elements, and/or (4) stable isotope ratios (SIRs) [34,35]. Additionally, the combined use of targeted and non-targeted metabolomics approaches to analyse polyphenols and other bioactive substances has also assisted in the classification of olive oils, especially in determining PDO [36].

Development of high-throughput analytical methods that can analyse samples in a fast and cost-effective manner will facilitate current certification systems and help meet the increased expectations of our stakeholders. The oil palm industry is committed toward building a supply chain that is traceable and transparent, which will promote sustainable practices in the industry. The industry’s actions are guided by the Sustainable Palm Oil Policy, which is based on current best practices informed by stakeholder input. The development of analytical techniques is also essential to determine if palm oil products are free from adulteration, i.e., presence of any foreign matter, in order to ensure its authenticity. While there are stringent specifications for palm oil that is traded, it is not uncommon for producers who do not meet the specifications to blend with other oils or batches of palm oil to meet the quality requirement for specific parameters. Another undesirable practice is adding sludge oil or lower quality to high quality oil to increase volume while complying with minimum specifications. This paper provides a review of the current technology applied to track vegetable oils across its supply chain and detect the presence of adulterants in crude palm oil (CPO). Early research being carried out to develop such methods for traceability and authenticity in the palm oil supply chain is described.

## 2. Traceability as Part of the Legislative Requirement for Regulating the Industry

Traceability has been defined by the General Food Law Regulation (2002) as “the ability to trace and follow food, feed, and ingredients through all stages of production, processing, and distribution” [19]. There is increasing pressure on exporting countries to understand and comply with new legislations introduced for traceability by importing countries, usually those with developed status. As such, there is a need to ensure that the traceability system in place is consistent with regulations of importing countries to avoid any misunderstandings and delays in shipments [37]. In recent years, enforcement to ensure traceability in the palm oil supply chain has been increasing and it is an important signal to the palm oil-producing countries on the importance of providing concrete data to importers, especially the customers in Europe. The intricacy of the supply chain makes it clear that traceability of palm oil and its products is not easily accomplished [38]. A robust traceability system is critical in ensuring that only sustainably-produced palm oil reaches the designated customers and will also easily resolve disagreements on the origin of the product. Oil palm by virtue of being very productive, will benefit most from a reliable traceability system. If certified sustainable palm oil (CSPO) can be effectively differentiated from non-CSPO palm oil along the supply chain, it will encourage the growing and trading of CSPO. CSPO that is traceable will likely increase acceptance of palm oil worldwide and can help meet the growing demand for oils and fats. Its high productivity means that the oil palm requires three to eight times less land area compared to other major oil crops. This directly translates to less clearing of forests relative to other oil crops for producing the same quantity of oil. Currently, it is planted on only 5% of the acreage dedicated to vegetable oils but contributes to 45% of global edible oil.

At present, traceability of palm oil products along the supply chain is conducted by inspecting written records via enforcement activities to help safeguard the oil palm industry from unscrupulous traders. The Malaysian palm oil supply chain has two levels of activities including upstream and downstream activities [39]. The upstream activities involve nursery operators, oil palm small-holders, fresh fruit bunch (FFB) dealers, and estates. Downstream activities are those associated with palm oil mills, refineries, and palm kernel crushing factories and also include export points. Engaging in these activities requires a license, a legal requirement under the MPOB (Licensing) Regulations [40], to avoid unhealthy and unwanted malpractices by businesses conducting palm oil-related activities. As a regulatory body, MPOB established a monitoring system aptly named “Palm Oil Monitoring System” in 2014 to regulate transfer of CPO from mills to refineries, as an important tracking system to ensure traceability. The palm oil transporter needs to be licensed and has to have a GPS system installed to ensure that the physical movement of palm oil is well monitored. Besides monitoring electronically, MPOB also carries out compliance checks for traceability by manually inspecting the document trails. All licensees are required to upkeep their daily trading record for enforcement inspection. Malaysia is looking at various options to strengthen its palm oil product traceability and authentication system to ensure a sustainable industry.

## 3. Analytical Methods for Traceability of Palm Oil Based on Geographical Origin

**Figure 1:**
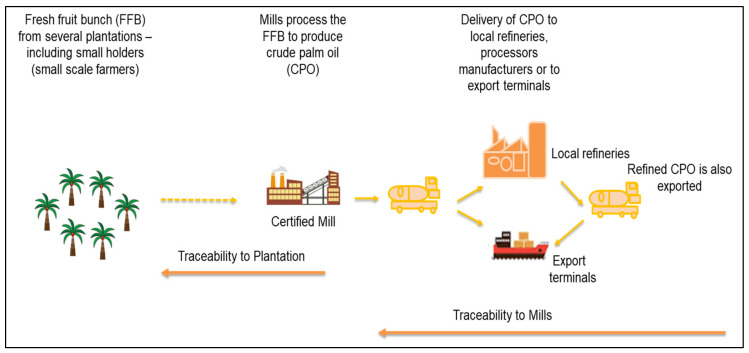
Scheme of palm oil traceability process from the plantation to the mill and export terminals.

Research carried out on traceability of edible oils examines both the botanical origin and/or the geographical region from where it has been sourced [41,42,43,44]. In both scenarios, identifying discriminating markers or compounds is typically complicated in light of the fact that the oil composition is determined by a multitude of interactive factors involving the genetic background of the planting material, environment in which it is planted, ripeness of the bunch at harvest, and oil extraction technology implemented in the mill. Step-wise geographical traceability across the supply chain of the oil palm industry relates to the ability to track and trace, which starts from the plantation in a specific geographical area to the mill where the CPO is produced and shipped to either refineries in Malaysia or to export terminals. The shipment of refined palm oil to the export terminals is also considered in the whole step-wise geographical traceability system as depicted in Figure 1.

Mill locations can indicate where the oil palm fresh fruit bunches (FFB) are processed and also provide information regarding which plantations supplied the mills [45]. Oil palm FFB must be processed immediately after harvesting, and no later than 24 hours, which suggests that the mills should ideally be located close to plantations. In Malaysia, mills are usually located in a 50-km radius from the plantation (however, this may differ depending on the accessible infrastructure, such as the quality and density of roads). This implies that analysing the geographical location of the mill and its surrounding infrastructure can reveal valuable data about plantations from which it sources its FFB. In addition to the standard paper documentation and electronic record maintained by the current certification system, other records that can verify geographical origin will provide corroborative evidence on the written information, which will have a huge positive impact on trading sustainable palm oil [26].

In order to determine the geographical origin of palm oil, researchers have been investigating and testing various analytical procedures with the hope to overcome the limitations imposed by the current traceability methods applied in the industry. Apart from traceability requirements for the certification procedures, a measure of the authenticity of samples must also be made by demonstrating the presence/absence of adulterants in comparison to standards or other samples of known quality. The methods used may incorporate multivariate statistical techniques to extract useful information (e.g., chemometric analysis) from a dataset.

### 3.1. Traceability of Palm Oil Using Chromatographic Techniques

Studies attempting to authenticate edible oils are known to have applied ‘chromatographic fingerprinting’ techniques [46], which provide unbiased information on the components present in the sample or extract. The use of multivariate data analysis will, thus, facilitate determination of the chemical characteristics of constituents in an extract and allows for the authentication and identification of a product. Edible oils and fats are routinely tested for fatty acid (FA) composition using gas liquid chromatography (GLC), and their purity can be determined by comparing the values obtained according to the guidelines established by the FAO/WHO Codex Committee on Fats and Oils [47]. There are a number of studies that demonstrate the advantages of applying various analytical tools for the classification of important vegetable oils based on their major components such as fats, sterols, proteins, and carbohydrates [48,49,50,51,52,53,54,55,56]. Table 3 provides an overview of the various methods applied for geographical determination of palm oil and its products, which will have an increasingly vital role moving forward. Geographical verification of palm oil was first reported using gas chromatography (GC) combined with a flame ionization detector (FID) while measurement of volatile organic compounds (VOCs) was achieved via proton transfer reaction-mass spectrometry (PTR-MS) [26]. The data was analysed using two binary Partial Least Squares Discriminant Analysis (PLS-DA) grouping models. First, a PLS-DA model was used to distinguish South East Asian palm oil samples from African and South American samples. Subsequently, a second model successfully separated the non-Asian samples or, more specifically, the African and South American samples. The ability to identify new and authentic samples further validated the models externally. This suggests that fingerprinting of fatty acids and volatile compounds in combination with chemometric analysis is a feasible approach for confirming the geographical origin of CPO.

The profiling of triacylglycerol (TAG) can be used as a tool to effectively identify vegetable oils and verify their compositions in order to detect any fraudulent practices and determine geographical origin [31]. The species-specific nature of the regulation of lipid biosynthetic pathways allows the assessment of TAG profiles to authenticate and differentiate vegetable oils. As such, adulteration, which involves the dilution of a commodity with less costly materials, can be detected [19]. For a particular vegetable oil crop, it may be conceivable to differentiate cultivars or the region used for cultivation based on a thorough investigation of the fatty acids (FAs). However, such a study could be tricky as it may require the analysis of the different combinations of FAs found on the TAG glycerol backbone.

The GC and HPLC chromatographic systems with appropriate detectors are routinely used in many laboratories for food analysis. These techniques, although somewhat selective and sensitive, do not provide clear information on the compound’s identity. The combined use of mass spectrometry (MS) and chromatographic techniques solves this shortcoming [54,56]. Lately, LC coupled to quadrupoles, magnetic sectors, or time-of-flight (TOF) detectors have made an impact on the analysis of food and its use can be expanded to determine its effectiveness in differentiating palm oil from different geographical areas. Two chromatographic techniques, namely HPLC, with a charged aerosol detector (CAD) and high temperature gas chromatography (HTGC)-mass spectrometry (MS) were effectively utilized to fingerprint the TAGs of CPO samples and develop appropriate authentication models [60]. The strategies were applied to profile palm oil samples sourced from three different regions/continents, namely South East Asia, Africa, and South America. The application of PLS-DA to analyse the TAG data could differentiate the samples based on their geographical origin with accuracy ranging from 70% to 100%.

Other than the study by Tres et al. [26], another interesting study that focused on the analyses of VOC, via the use of chromatographic and spectroscopic techniques, was by Ruiz-Samblas et al. [60]. The study employed proton transfer reaction mass spectrometer (PTR-MS) combined with two consecutive discriminant analysis (PLS-DA) models to analyse VOC of CPO samples from various geographical regions. The analyses of VOC could differentiate the African and South American samples with 100% accuracy, while the South East Asian samples were discriminated from the rest with approximately 88% precision. In a recent study [19], a relatively new innovation—GC-ion mobility spectrometry (GC-IMS)—was applied to investigate palm oil sourced from various countries, where differences observed in the VOC profiles could potentially support the existing administrative controls applied in the supply chain. IMS coupled with other methods, namely GC and LC, have been successfully applied for olive oil, where there was an increase in the sensitivity and selectivity of the resulting analysis [62]. The results, thus, far have demonstrated the potential of GC-IMS as a fast and cost-effective method to analyse VOC profiles of palm oil sourced from different countries across three continents [19]. Analysis of olive oil samples has revealed that the presence of certain molecules, elements, or volatiles in the oil is reflective of the soil, environment, and the production process, which allows for the differentiation of olive oil based on its region of origin. However, similar research in palm oil is still in its infancy. The discrimination of samples originating from different species and/or different geographical origins is based on their different chemical compositions. Nevertheless, there are exceptions where samples from a particular location can contain different components, while the chemical composition of samples obtained from different areas may be the same [63].

### 3.2. Analysis of a Stable Isotope as a Traceability System for Palm Oil

The best strategy to precisely determine the geographical origin of a product is by measuring the concentration of elements and the variation of isotopes in the product. Such methods have been utilized to determine the geographical origin of a wide range of products including food with some degree of confidence [35,64,65,66,67,68,69]. The investigations look at measuring the isotope ratios of carbon and nitrogen to obtain information on selected plants while the isotope ratios of oxygen, hydrogen, sulphur, and strontium can assist in revealing the place of origin of a particular product. As such, this approach is extremely important and likely applicable to palm oil traceability, provided there is access to the instrumentation required for the analysis. Muhammad et al. [27] utilised the isotope ratio mass spectrometer (IRMS) to analyse specific stable isotopes (δ^13^C, δ^2^H, and δ^18^O) in CPO samples that were obtained from different states in Malaysia. By combining the three isotopic ratios and applying a hierarchical cluster analysis, 33 samples of CPO were assigned to the respective states in Malaysia from where they originated with a predictive power of 52.60%. The study also concluded that δ^18^O is the most promising marker for discriminating the samples randomly collected from across Malaysia. Furthermore, in another preliminary study, the IRMS method was subsequently used to analyse CPO samples obtained from four mills representing three zones in Peninsular Malaysia (North, South, and Central regions) and Sarawak region, respectively [61]. As shown in Table 4, ANOVA showed no significant differences within groups, i.e., (MXPn_1-10_). However, multiple comparison tests (Tukey and Duncan tests) could differentiate palm oil from mill 1 (M1Pn_1-10_) and mill 3 (M3Pn_1-10_) from the other groups. However, no differentiation could be made between palm oil from mills 2 (M2Pn_1-10_) and 4 (MPn_1-10_). Moving forward with techniques like IRMS, apart from the cost of the instrumentation, there should be a clear realization that large number of samples have to be analysed to draw more precise and definitive conclusions on the origin of samples [70]. However, the main drawback is the cost of purchasing and maintaining some of the required instrumentation with the additional need for specialized personnel to run the instruments [70]. Strong data analysis support to correctly classify products based on place of origin is also necessary.

In the long-term, we hope that development of more robust methods to detect stable isotopes and chemical signatures will allow such techniques to be incorporated into palm oil traceability systems to better protect the supply chain. The research described by Ramli et al. [61] is in the early stages, and the investigators themselves recognise the limitations of the small sample size in accurately determining geographical origin. Therefore, there is a need for a more comprehensive analysis to verify the homogeneity of the results and establish appropriate benchmarks to assist scientific developments in the near future.

Crops are typically grown on soils, and the soil maps of countries differ. This fact influences the concentration of specific elements in the final products including food, which can facilitate the assignment of geographical origin. As such, there has to be careful consideration of the appropriate choice of analytical methods. Each analytical approach has certain advantages and also shortfalls. Among the most commonly used techniques for food authenticity and traceability, spectroscopic techniques coupled with appropriate data analysis tools, particularly chemometrics, are more attractive and promising compared to chromatographic techniques to successfully discriminate the samples.

## 4. Analytical Methods for Detecting Adulterations in Palm Oil

Palm oil quality is dictated by a few parameters such as free fatty acid content, peroxide value, moisture, and impurities. Malaysia has strict practices in ensuring the quality of its CPO, where the typical Malaysian palm oil consists of more than 90% triacylglycerols, 3–8% diacylglycerols, and about 1% minor components such as vitamin E (tocopherols and tocotrienols), sterols, carotenoids, phospholipids, glycolipids, terpenic, and aliphatic hydrocarbons [2,71,72]. The levels of monoacylglycerols and free fatty acids (FFA) is kept below 3% and 3–4%, respectively [71,72]. MPOB standards stipulate a maximum allowable limit of 5% FFA, while moisture content and impurities should be capped at 0.25% in the CPO [73]. The Palm Oil Refiners Association of Malaysia (PORAM) recommends that FFA in palm oil should be kept below 0.1% [74].

There is constant development of advanced techniques to detect adulteration of edible oils such as those based on HPLC-ESI-MS fingerprinting. These techniques are being applied to certify edible oils to ensure that there is no blending with other oil sources (unless specifically indicated) and/or with used cooking oil [75,76,77]. The advantage of the techniques is their ease of use and performance, and the individual analysis can be completed in a relatively short time. One of the earlier analysis to differentiate different edible oils based on their fatty acid profiles was reported by Zhang et al. [78]. In this study, they profiled the fatty acids from five kinds of edible oils using gas chromatography combined with mass spectrometry (GC/MS) in a selected ion monitoring mode. A total of 28 fatty acids was identified using mass spectral characteristics of selected ions and equivalent chain length (ECL). The classification of the vegetable oil samples was achieved using supervised and unsupervised multivariate statistical analysis methods, which incorporated principal component analysis, hierarchical clustering, and random forest analysis. The results demonstrated the classification of the five vegetable oil samples to five distinct groups, which proved to be useful for an authenticity assessment.

Research reports describing the use of analytical methods for assessing palm oil adulteration, either during palm oil production or food processing, are tabulated in Table 5. One of the growing concerns of the oil palm industry is food safety, where there are incidences of palm oil adulteration with residual oils such as sludge palm oil (SPO) and palm fibre oil (PFO). These residual oils are sometimes added back to the CPO to expand the volume obtained in a particular mill [79,80]. The acidic nature of SPO makes it unsuitable for food applications, and the practice of adding it to the CPO is undesirable as it will compromise the quality of palm oil [81]. PFO is obtained from pressed palm fruits that have gone through one round of oil extraction via the use of solvents. The nutrient rich PFO is usually added back to CPO to maximise oil extraction rates. However, this practice is no longer recommended, as it can likely enhance build-up of 3-monochloropropane-1,2-diol esters (3-MCPDE), which are undesirable in the CPO [82]. Most of the research carried out to detect residual oils or other unwanted residues in CPO has involved the blending of sludge oils (SO) and used vegetable oils (UVO) into CPO at a range of 1 to 20% (*v*/*v*).

**Figure 2:**
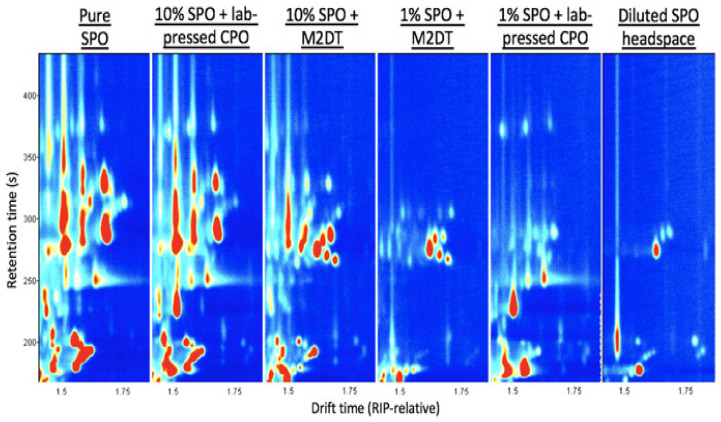
Reporter view showing the sludge palm oil (SPO) fingerprint across a range of samples. 10% SPO + lab-pressed crude palm oil (CPO) and 10% SPO + Mill 2 Dispatch Tank (M2DT) are most similar to the pure SPO fingerprint. 1% SPO + M2DT CPO and 1% SPO + lab-pressed CPO share similarities with both pure SPO and diluted SPO headspace [83].

Othman et al. [83] reported the use of GC-IMS to analyse CPO samples for the presence of the undesirable residual oils. This was carried-out using CPO that was extracted in the lab, where SPO and PFO was intentionally added to the CPO at concentrations of 1% and 10% (*w*/*w*), respectively. The spectrum obtained was then compared with that of un-spiked CPO. A total of 16 signals unique to PFO were identified of which just seven were detected in the lab extracted CPO spiked with PFO. Four of the signals were detected at a 1% (*w*/*w*) level, while the remaining three were detected at a 10% (*w*/*w*) level. Similarly, to determine the ability of GC-IMS to detect SPO fingerprints, four different SPO concentrations were spiked into both lab-pressed CPO and CPO collected from mills located in four different geographical areas in Malaysia. The results are presented in Figure 2. The SPO fingerprints could be easily distinguished from both the lab-pressed CPO and CPO collected from four different mills. The SPO fingerprints when spiked into the CPO of the four different mills were also similar and were not influenced by the variability in the source of the CPO. More importantly, similar SPO-related fingerprints were observed for all four concentrations tested, which ranged from 1% up to 10%. The results suggest that SPO can be easily detected if blended into CPO, and the detection limit is 1% SPO, or possibly lower. This was the first report detailing the use of GC–IMS for fast, cost-effective analysis of CPO for the presence of volatile organic compound (VOC) fingerprints due to inclusion of PFO and SPO. The results also suggest GC-IMS is a reliable tool for detecting specific SPO VOC fingerprints, and can be used for rapid screening on-site. Similar to this study, Hamdan et al. [79] detected SPO at low concentrations using a different approach, which exploits the dielectric properties of CPO at different temperatures and sludge contamination levels. The applicability of GC-IMS in different areas of food analysis, authentication, adulteration, and safety have been demonstrated in many different studies [84,85,86,87]. The advantages attributed to GC-IMS can be further enhanced if integrated with additional separation technologies such as traditional liquid chromatography (LC) and gas chromatography (GC) mass spectrometry (MS) workflows to identify specific compounds with added confidence, either in a targeted or non-targeted approach.

More recently, adulteration of fresh cooking oil with used cooking oil has raised concerns in Malaysia with respect to food safety. FTIR spectral analysis and investigation of fatty acid composition (FAC) profiles reportedly showed clear differences between fresh palm olein, used cooking oil, and adulterated oils [88,89]. The techniques employed could detect adulteration of fresh cooking oil with used cooking oil with a high degree of confidence. Nevertheless, newer and more efficient methods to determine adulteration of lard in fats, oils, and fat-based products is required to safeguard consumers and the food-based industries. The position-specific distributions of fatty acids on the glycerol molecules of triglycerides are the ideal fingerprints for characterising vegetable oils.

A combination of chemical, physical, and/or chromatographic methods has been applied to evaluate the triacylglycerol composition of edible oils, as a strategy for evaluating the presence of adulterants in the oils [95]. Since there is an increasing number of adulterants, limiting the analysis to fatty acid profiles is likely not effective enough for verification purposes. Therefore, it is essential that components like phytosterols and non-saponifiable compounds present in some vegetable oils can also be more frequently used as markers for the detection of adulterated oils [96,97]. However, extraction of these markers is tedious and challenging, and point to an interesting area of future research.

## 5. DNA Analysis as a Tool for Traceability and Authenticity

Since the chemical composition (including ratio of stable isotopes) of vegetable oils can vary, depending on several factors, which include the growing season and processing techniques employed, alternative methods are also being explored to assess traceability and authenticity of edible oils [98]. The use of DNA analysis is proving to be popular, especially since the genomes of a large number of vegetable oil crops have been decoded, and DNA fingerprints are generally independent of environmental variations. Traceability using genetic (DNA) analysis is especially prevalent for virgin olive oil that is produced using a mechanical press without the use of any chemical extraction procedures. DNA analysis in such cases using both nuclear [99] and chloroplast DNA [100] markers is able to distinguish, with some success, the monovarietal olive oil that has been labelled as “protected designation of origin (PDO)” by the EU. In fact, in some instances, the use of DNA analysis was more effective than GC analysis of fatty acid profiles to detect adulteration of olive oil [101]. However, Crawford et al. [102] opined that fatty acid profiling coupled with multivariate analysis was more effective than DNA analysis in differentiating olive cultivars. The contrasting results indicate that establishing reliable tools for traceability and authenticity analysis is still an important active area of research where a consensus on the most effective method(s) will take time to develop. The main challenge in using DNA markers has been establishing a DNA extraction and analysis method, mostly based on polymerase chain reaction (PCR), which, even now for olive oil, is being improved for more effective analysis of the commodity [103]. The use of DNA analysis is more complicated for some vegetable oils (e.g., palm oil) that go through a complex milling and refining process involving high temperatures, which can cause extensive fragmentation of the DNA and compromise its amplification in a PCR assay. Nevertheless, early success has been reported for some of these, namely Camellia oil that is rich in unsaturated fatty acids [104]. In this study, DNA analysis successfully detected adulteration of the Camellia oil, which was not possible via GC analysis of fatty acids [104]. In the case of palm oil, Ooi et al. [105] found it challenging to extract good quality DNA that was free of impurities from CPO. However, Zhang et al. [106] established a protocol for extracting DNA from commercially-available palm oil and successfully amplified the low copy *MT3-B* gene (a metallothionein gene), which was supposedly palm oil-specific. The researchers detected the presence of palm oil when blended with soybean oil using PCR analysis of the *MT3-B* gene, which indicated its potential for authentication of both palm and soybean oil.

The use of DNA-based methods has had more success in olive oil for both traceability to specific geographical origin and for authenticity purposes. Similarly, DNA-based analysis has also had some success when applied to generate unique fingerprints for cold-pressed hazel nut oil [107]. However, the use of DNA markers in other vegetable oils, which undergo more rigorous milling and refining processes, has mostly focused on detecting adulterants or to differentiate genetically and non-genetically modified crops [108]. The ability of DNA markers to track edible oils across the supply chain to their geographical origin is still at the research phase. The use of DNA markers for this purpose, especially for a crop like oil palm, is going to be challenging, as genotypes with different genetic backgrounds are usually planted, even in a single block of a large plantation. The fact that the fruit bunches that give rise to the CPO in commercial estates result from open pollination, often from various sources of pollen (even within a single bunch), may add to the complications. Nevertheless, traceability requires the use of a wide repertoire of technologies to obtain sufficient resolution to effectively track a sample across the supply chain [109]. As such, DNA analysis in combination with other analytical techniques, where the data is analysed in a multi-dimensional approach, is likely required in the future to successfully track palm and other vegetable oils across their supply chain.

## 6. Conclusions

Geographical determination schemes are expanding worldwide. The ability to classify palm oil based on its geographical origin and authenticate its purity using new state-of-the-art techniques is vital to support the current certification procedures, which are largely based on available documentation. Currently, the combined use of advanced analytical techniques and chemometrics has been successfully applied to determine the geographical origin of edible oils, and these available techniques should be exploited by the oil palm industry. Currently, many laboratories have a repertoire of analytical equipment, which allow many data-fusion strategies to be implemented in multivariate approaches. The availability of highly precise analytical fingerprinting procedures for authentication of palm oil and for determining its geographical origin for certification purposes is a real and important challenge facing the industry. Considering the importance of protecting consumers and reliable producers as well as the importance of assisting law enforcement activities, it is imperative that the industry develops systematic analytical techniques for measuring authenticity of the palm oil consignment, verify its place of origin, and make sure that it is free of adulterants.

## Figures and Tables

**Table 1:** Identity characteristics and quality specification of Malaysian crude palm oil (CPO).

Identity Characteristics	Range	Mean
Apparent density, kg litre^−1^, at 50 °C	0.8890 to 0.8950	0.89050 ± 0.0002
Refractive Index, n_D_ 50 °C	1.454 to 1.456	1.4543 ± 0.0002
Saponification value, mg KOH g^−1^ oil	194 to 205	199 ± 2.4
Unsaponifiable matter, %	0.19 to 0.44	0.32 ± 0.066
Fatty acid composition, (wt % as methyl esters)		
C12:0	0.0 to 0.5	0.2 ± 0.10
C14:0	0.9 to 1.5	1.1 ± 0.08
C16:0	39.2 to 45.8	43.5 ± 0.95
C16:1	0.0 to 0.4	0.2 ± 0.05
C18:0	3.7 to 5.1	4.3 ± 0.18
C18:1	37.4 to 44.1	39.8 ± 0.94
C18:2	8.7 to 12.5	10.3 ± 0.56
C18:3	0.0 to 0.6	0.3 ± 0.07
C20:0	0.0 to 0.5	0.2 ± 0.16
Iodine value (Wijs)	50.4 to 53.7	52.0 ± 0.66
Slip melting point, °C	33.8 to 39.2	36.7 ± 0.84
Total carotenoids (as β-carotene), mg kg^−1^	474 to 689	581 ± 45.5
**Quality Characteristics**	**Special Quality CPO**	**Standard Quality CPO**
Free fatty acid (as palmitic), % max	2.5	5.0
Moisture and impurities, % max	0.25	0.25
Peroxide value, meq O_2_ kg^−1^ max	1.0	2.0
Anisidine value, max	4.0	5.0
DOBI, min	2.8	2.3

Source: Bustamam et al. [4].

**Table 2:** Malaysian oil palm industry performance.

Performance Indicators	2019	2018	Difference	
			Vol./Value	%
Opening stocks (mil tonnes)	3.22	2.73	0.48	17.7
CPO production (mil tonnes)	19.86	19.52	0.34	1.8
FFB yield (t ha^−1^)	17.19	17.16	0.03	0.2
Oil extraction rate (%)	20.21	19.95	0.26	1.3
PO exports (mil tonnes)	18.47	16.49	1.98	12.0
PO imports (mil tonnes)	0.98	0.84	0.14	16.1
Closing stocks (mil tonnes)	2.01	3.22	(1.21)	(37.5)
CPO price (RM t^−1^)	2,079.00	2,232.50	(153.50)	(6.9)
Export revenue (RM billion)	64.84	67.52	(2.68)	(4.0)

Note: CPO—crude palm oil, FFB—fresh fruit bunch, PO—palm oil, mil tonnes—million tonnes, Vol.—Volume. Source: Malaysian Oil Palm Statistics [15].

**Table 3:** Summary of relevant publications describing analytical instruments and chemometric analysis of palm oil to determine geographical origin.

Parameters Measured	Analytical Methods	Data Interpretation	Summary and Main Findings	Advantages	Limitations	Reference
Chromatographic Approaches						
TAG	HPLC-DAD	SIMCA and PLS-DA	Margarines and fat spread products were successfully differentiated based on their region of origin (Spain and Morocco) using UV-absorption fingerprints of unsaturated fatty acid proportion of TAGs and multivariate chemometric tools.	Pre-treatment of samples prior to chromatographic analysis is not required. Samples only need to be dissolved in n-hexane prior to analysis.	Samples from Europe are similar to those from Spain and were not differentiated by the model.	[31]
FAC and VOC	GC-FID and PTR-MS	PLS-DA	Analysis of FAs/VOCs by GC-FID/PTR-MS combined with chemometrics was a feasible approach to separate CPO by continent. Saturated FAs such as C12:0, C14:0, and C16:0 were higher in CPO from South East Asia while South American samples contained higher monounsaturated FAs from the n-9 series, such as C18:1n-9 and C20:1n-9. Polyene chain of some carotenoids and their degradation compounds, furfural, or heptanal were proposed as VOC markers for regional classification.	PTR-MS is sensitive with low fragmentation. VOC in the sample head-space are detected without prior chromatographic separation, and non-conformities that would be unnoticed using the traditional targeted approach are detected. GC-FID is a reliable standard method in FA analysis and can successfully discriminate samples based on the fatty acid profile.	PTR-MS is not very effective in identifying the VOC compounds as it is essentially a low-resolution one-dimensional technique. PTR-MS hardly measures less volatile VOC components such as carotenoids, which could also be degraded during palm oil extraction and storage.	[26]
K extinction values (K_270_, K_232)_, colour, chlorophyll, carotenoids content	UV-visible spectroscopy	PCA and OPLS-DA	UV-visible spectroscopy coupled with quality characteristic assessment proved effective in discriminating 60 CPO samples from North, South, and Central Nigeria based on spectral (K_270_, K_232_, colour, chlorophyll, and carotenoids) and chemical (FFA, acidity, and PV) assessment. Central Nigeria CPO samples were distinguished by significantly high carotene content, free fatty acids, acid value, and peroxide value while North Nigeria samples were separated by K extinction values, colour density, and chlorophyll content.	UV-visible spectroscopy is cost-effective and one of the less complicated techniques in food analysis. It has shorter analysis time and small sample requirement. This approach could also be a valid tool to avoid misrepresentation of CPO quality.	Requirement for a large sample size to identify differences and distinguish samples based on the region of origin.	[57]
Chromatographic data fusion	HPLC-UV and HPLC-CAD	PLS-DA	Geographical authentication of 100 CPO samples obtained from diverse regions (South-East Asia, West Africa, and South America) was achieved by the analysis of FAME, phytosterols, and terpene alcohols, using two different analytical tools combined with the PLS-DA classification approach.	Improved classification and high accuracy of the model (ranging from 87% to 100%) were achieved by applying data fusion strategies to combine the data from HPLC-UV and HPLC-CAD.	Additional cost due to the use of multiple platforms to analyse the samples.	[58]
Sterolic chromatographic data	HPLC	SIMCA and PLS-DA	Sterolic chromatography was used to discern the geographical origin of 102 CPO samples from South-East Asia, West Africa, and South America. Sterolic chromatographic profiles for African CPO did not show any unique pattern where some samples were similar to those obtained from South America and South-East Asia. This is likely due to the common origin of the commercially planted oil palm in the three regions.	Application of HPLC with properly optimised chromatographic conditions and suitable detector can be used to generate fingerprint data for differentiating samples.	None of the tested chemometric tools were sensitive enough to distinguish samples based on their region of origin. There was still up to 15% inaccurate classification of samples, even when using the best system implemented in the study.	[59]
TAG	HPLC–CAD and HTGC–MS	PLS-DA	TAG fingerprinting measured by HPLC-CAD and HTGC-MS was applied to describe the origin of palm oil from three main palm oil producing regions, Southeast Asia, West Africa, and South America. A similar chemometric process employing PLS-DA was used on both types of chromatographic data and their results were compared. The authors concluded that the methods provide a rapid tool for palm oil classification to verify labelling compliance of geographical origin.	Both chromatographic methods (HPLC-CAD and HTGC-MS) provide a significant advantage over conventional LC and GC as they only involve sample dilution without additional sample treatment or derivatization procedures prior to injection. The use of a different detector such as CAD also improves acquisition time.	FA composition of TAGs was not elucidated. PLS-DA model from HTGC-MS data show low specificity of 75% for South East Asian samples.	[60]
Stable Isotope Approach						
Stable carbon (δ^13^C), hydrogen (δ^2^H), and oxygen (δ^18^O) isotopic compositions	EA- and TC/EA-IRMS	HCA, PCA, and OPLS-DA.	The first report that used stable isotope profiles to study traceability of CPO samples to its origin. The study evaluated samples from different regions in Malaysia.	Possible to predict the origin of CPO samples at a narrower geographical area, i.e., based on regions in a specific country	Accessibility to instrument is limited since its availability is not as widespread as other hyphenated mass spectrometers	[27]
Stable δ^13^C	EA-IRMS	ANOVA and Multiple comparison tests (Tukey and Duncan tests)	The preliminary study revealed that specific chemical fingerprints related to the carbon isotope could be detected in the CPO samples, which reveals their potential for application in a traceability system.	Possible to differentiate the origin of CPO samples produced from different palm oil mills likely due to the different milling practices	Geographical origin discrimination may be difficult when the areas in question have similar climate. However, other factors such as agricultural regime and milling practice can come into play for a successful discrimination. As such, there is a need to analyse multiple stable isotopes instead of depending on a single element	[61]
VOC Fingerprinting						
VOC fingerprints	GC-IMS	Chemometrics	The potential utility of GC-IMS as a fast monitoring technology of VOC fingerprints of palm oil sourced from various continents/sub-continents (Malaysia, Africa, and South America) was demonstrated. VOC fingerprints that are only present in palm oil produced in Colombia were reported, which could be used as rapid markers for visualizing similarities and differences in VOC composition of different samples.	The higher resolved 3D fingerprints obtained from the GC-IMS system provide superior resolving power for non-targeted profiling of VOC fractions from highly complex samples such as palm oil. IMS can also be coupled to other techniques such as GC or LC to increase sensitivity and selectivity. This facilitates its application for preliminary screening of oils in the field as well as in larger analytical laboratories.	GC-IMS is not suitable for identifying unknown compounds. Hyphenation with other powerful technologies is required to identify individual VOC components.	[19]

Note: CPO—crude palm oil. TAG—triacylglycerol. VOC—volatile organic compound. FFA—free fatty acid. FAC—fatty acid composition. PLS-DA—partial least squares-discriminant analysis. GC-FID—gas chromatography-flame ionization detector. PTR-MS—proton transfer reaction-mass spectrometry. PCA—principal component analysis. SIMCA—soft independent modelling of class analogy. HCA—hierarchical cluster analysis. OPLS-DA—orthogonal partial least squares-discriminant analysis. PV—peroxide value. K_270_—conjugated triene value. K_232_—conjugated diene value. HPLC-UV—high-performance liquid-chromatography coupled with an ultraviolet detector. HPLC-CAD—high-performance liquid-chromatography coupled with charged aerosol detector. HPLC-DAD—high-performance liquid-chromatography coupled with a diode array detector. HTGC-MS—high temperature gas chromatography-mass spectrometry. EA—elemental analyser. TC/EA-IRMS—Thermo Finnigan elemental analyser coupled to an isotope ratio mass spectrometer. ANOVA—analysis of variance. GC-IMS—gas chromatography-ion mobility spectrometry.

**Table 4:** Measurement of stable carbon isotope ratio.

Sample ID		N	Subset for Alpha = 0.05		
			1	2	3
Tukey HSD^a^	M1Pn	10	−31.088110		
	M2Pn	10		−30.890524	
	M4Pn	10		−30.832998	
	M3Pn	10			−30.692361
	Sig		1.000	0.481	1.000
Duncan^a^	M1Pn	10	−31.088110		
	M2Pn	10		−30.890524	
	M4Pn	10		−30.832998	
	M3Pn	10			−30.692361
	Sig		1.000	0.157	1.000

M: Mill. Total number of CPO = 10. a: Uses Harmonic Mean Sample Size = 10.000 [61].

**Table 5:** Recent publications on analytical methods for detecting adulteration of palm oil.

Study Case	Parameters Measured	Analytical Methods	Data Interpretation	Summary and Main Findings	Advantages	Limitations	Reference
Adulteration of crude palm oil with secondary and used cooking oils							
Adulteration of CPO with PFO and SPO	VOC	GC-IMS	Topographical plot	The paper reports on the assessment of GC–IMS for rapid screening of VOC fingerprints of secondary oils such as PFO and SPO manually added into CPO. VOC fingerprints of SPO were distinguished at 1% to 10% concentration. Seven PFO and 21 SPO markers were effectively detected.	In comparison to classical techniques, IMS coupled to GC is potentially a more rapid, accurate, and cheaper analytical system to discriminate different types of edible oil samples. In addition, it may assist in developing appropriate references for grade identification for use by vegetable oil refineries.	Clear differences between samples could be observed. However, it was difficult to realize digital expression. Therefore, it was necessary to apply appropriate chemometric tools to establish a recognition model.	[83]
Adulteration of CPO with SPO and used UVO	FAC, TAG	GC-FID and HPLC-ELSD	PCA and CO	The combination of chemical properties and multivariate analysis was applied to detect differences between CPO and CPO blended with SPO or UVO. Detection of concentrations of 5% and 2% (v/v) was achieved for SPO and UVO based on FAC (GC-FID) and TAG (HPLC-ELSD) analysis	Potential to differentiate adulterated CPO with SPO and UVO.	Depending solely on FAC and TAG for distinguishing pure and adulterated CPO is not ideal due to the dominant effect and similar chemical properties of CPO and adulterant SPO and UVO. Detection level of the adulterants was relatively low and changes in the chromatograms were not very significant.	[90]
Adulteration of CPO with SPO	Storage and dissipation of electric and magnetic energy in oil	Dielectric spectroscopy	PCR and PLS	Significant differences between the dielectric constants of pure CPO and contaminated CPO (*p* < 0.01) were observed. The results clearly showed that the dielectric constant of contaminated CPO increased with the incorporation of sludge oil from 0.6% to 10%. This was likely due to the high moisture content in SPO, which further induced its polarity and coincided with the dielectric constant value.	Dielectric spectroscopy technique is a rapid analysis system. Furthermore, the non-destructive nature of the analysis makes this an interesting alternative tool to enhance the efficiency of monitoring palm oil quality.	Dielectric spectroscopy is considered as a relatively new technique. Its efficiency in effectively extracting valuable information for application in the field of food quality and authentication of palm oil has not been fully verified.	[79]
Adulteration of processed palm oil with used cooking oil							
Adulteration of RFO	Raman spectroscopy	Raman microscope	PCA	Raman microscope and PCA were used to detect adulterated and unadulterated frying oils. Spiked samples were prepared by adding recycled cooking oils (heated-palm cooking oil, fried-pork oil, fried-chicken oil, fried-dory fish oil, and fried-banana oil).	Raman spectroscopy is cost-effective, non-destructive, fast, and convenient and requires little to no sample pre-treatment.	Raman spectroscopy is not able to quantify the actual percentage or level of adulteration in the spiked samples. It can only detect that adulteration has occurred	[91]
Adulteration of RCO	FAC and FTIR	GC-FID and FTIR spectroscopy		The use of the analytical systems could differentiate pure and adulterated PO at 100% accuracy, even at very low adulterant concentration (1%). The FTIR data combined with PLS-DA showed extremely good performance (coefficient of determination, R^2^ = 0.995). The level of polyunsaturated fatty acids (PUFAs) decreased with the increase in adulteration, which by itself is a good indicator for adulteration with RCO.The findings from this study provides useful information to regulatory authorities in constructing standard guidelines for the detection of cooking oil adulteration.	Both analytical techniques are rapid, reliable, and accurate. They are easy to use and require minimal or no sample preparation.	Both techniques applied needed refinement with regard to principles and concepts in order to achieve the conclusive outcome of detecting adulteration of PO with RCO.	[88]
Adulteration of UCO	Mid-IR (MIR) and NIR spectrum	FTIR and FT-NIR	DA	The techniques were successful for authenticity screening with 96.7% correct classification on FT-IR and 83.3% correct classification on FT-NIR. Further improvements can be made for the spectroscopy techniques for routine application to authenticate and assure quality of edible palm oil in the future.	MIR and NIR spectroscopy is fast, sensitive, reliable, and non-destructive. In addition, it is relatively easy to operate and there is almost no sample preparation. It is cost-effective and environmentally-friendly.	Cannot separate individual components/adulterants but only identifies adulteration.Data analysis and interpretation is not simple, and requires chemometrics.	[89]
Adulteration of PPO with animal fats							
Adulteration frying oil with lard	FTIR spectraand FAC	FTIR and GC	PLS	FTIR spectroscopy analysis in combination with multivariate calibration of PLS-DA was effectively used for detecting the presence of lard in pre-fried French fries. The result indicated that lard in a mixture of PO can be detected even at 0.5% level. PLS-DA model gave a linear regression with good coefficient of determination (R^2^ = 0.9791), which reflects good prediction of FTIR spectra against the actual level of lard. The FAC was used to verify the spectral band width. Unlike PO, lard contains two fold higher linolenic acyl groups than PO.	The simplicity, relatively fast analysis, and high accuracy of FTIR spectra method will facilitate its incorporation for routine quality control analysis. All PO samples adulterated with lard were clearly differentiated from non-adulterated PO.	Not applicable	[92]
Adulteration of PPO with lard	Electric nose	A zNose vapour analysis system	Analysis of variance	Surface acoustic wave sensing electronic nose enabled the detection of lard in refined, bleached, deodorized palm olein at levels as low as 1%. There was higher concentration of VOC for lard compared to olein. Results were compared to GC analysis of FAME, which found that C15:0, C17:0, and C17:1 fatty acids were unique to lard.	Electronic nose is regarded as a green technique in this field, which makes it ideal for quality control purposes. It is non-destructive, rapid, and relatively low cost.	Method may not be applicable for adulteration levels ≤1%	[93]
Adulteration of RBD PO with lipase-catalysed inter esterified lard	TAG, FAC, DSC	GLC, RP-HPLC, and DSC	Step-wise multiple linear regression	The presence of lipase-catalysed interesterified lard in RBD palm olein was examined using GLC, RP-HPLC, and DSC. Among the methods employed, DSC was most effective at detecting lard using its thermal characteristics, even at 1% levels.	DSC was more sensitive for both quantitative and qualitative determination of ERLD in palm oil. In addition to the accuracy and speed, the DSC method also provides a better means of identification of lard with a detection limit of 1%.	Applications of HPLC and GLC were less effective for qualitative and quantitative analysis of adulterants such as ERLD. Both analytical instruments were not able to distinguish between PO adulterated with lard and those adulterated with chicken fat.	[94]

Note: CPO—crude palm oil. PFO—palm fibre oil. SPO—sludge palm oil. VOC—volatile organic compound. GC-IMS—gas chromatography-ion mobility spectrometry. UVO—used vegetable oil. RCO—recycled cooking oil. RFO—recycled frying oil. UCO—used cooking oil. FAC—fatty acid composition. TAG—triacylglycerol. GC-FID—gas chromatography-flame ionization detector. HPLC-ELSD—HPLC with evaporative light scattering detector. FTIR—Fourier transform infrared spectroscopy. FT-NIR—Fourier transform near-infrared spectroscopy. PPO—processed palm oil. DSC—differential scanning calorimetry. ERLD—enzymatically-randomized lard. GLC—gas liquid chromatography. RP-HPLC—reversed phase-high performance liquid chromatography. PCA—principal component analysis. CO—cluster observation. DA —discriminant analysis. PCR—principal component regression. PLS—partial least squares.

## Patent

Noor Idayu Mhd Tahir, Umi Salamah Ramli, Abrizah Othman, Rajinder Singh, Nor Hayati Muhammad, Ahmad Parveez Ghulam Kadir, and Ravigadevi Sambanthamurthi (2020). Method for detecting adulterants in crude palm oil (CPO). PI2020001766.
